# Draft Genome Sequence of the Nominated Type Strain of “*Ferrovum myxofaciens*,” an Acidophilic, Iron-Oxidizing Betaproteobacterium

**DOI:** 10.1128/genomeA.00834-14

**Published:** 2014-08-21

**Authors:** Ana Moya-Beltrán, Juan Pablo Cárdenas, Paulo C. Covarrubias, Francisco Issotta, Francisco J. Ossandon, Barry M. Grail, David S. Holmes, Raquel Quatrini, D. Barrie Johnson

**Affiliations:** a Fundación Ciencia & Vida, Santiago, Chile; b Facultad de Ciencias Biologicas, Universidad Andres Bello, Santiago, Chile; c College of Natural Sciences, Bangor University, Bangor, United Kingdom

## Abstract

“*Ferrovum myxofaciens*” is an iron-oxidizing betaproteobacterium with widespread distribution in acidic low-temperature environments, such as acid mine drainage streams. Here, we describe the genomic features of this novel acidophile and investigate the relevant metabolic pathways that enable its survival in these environments.

## GENOME ANNOUNCEMENT

“*Ferrovum myxofaciens*” is an obligately aerobic, chemolithoautotrophic, and psychrotolerant acidophile that grows only by oxidizing ferrous iron (1, 2). Although “*Ferrovum*”-like bacteria have been identified in the clone libraries of acid mine waters from many different locations, isolate P3G (the designated type strain) is the only reported representative of this genus (and of the proposed new bacterial order “*Ferrovales*”) grown in axenic culture (2). “*Ferrovum myxofaciens*” P3G was isolated from a stream draining an abandoned copper mine in Wales, where it formed macroscopic acid streamers (1, 3). “*F. myxofaciens*” P3G and the related clone sequences form a monophyletic group within the *Betaproteobacteria* distantly related to classified orders (2).

The genome of strain P3G was sequenced using the Illumina MiSeq platform and paired-end libraries with insert sizes of ~500 bp. Quality-filtered reads (4) were assembled *de novo* using Velvet (version 1.2.10) and a *k*-mer length of 127 (5). The draft genome size is 2.7 Mb, with a median coverage depth of 83-fold and an average G+C content of 54.9%. It contains 173 large contigs (>1,000 bp), with an *N*_50_ of 32,564, and 473 smaller contigs. Genes were identified using previously reported annotation pipelines and manual curation (6). The draft genome annotation predicts 46 tRNA sequences, 1 rRNA operon, and 2,648 protein-coding genes, 36.8% of which were assigned putative functions (7).

As with most other sequenced acidophilic autotrophs, the complete set of genes for Calvin-Benson-Bassham CO_2_ fixation were identified, including genes for carboxysome formation. The draft genome also contains the full set of *nif* genes required for nitrogenase assembly and maturation, including the molybdenum transport cluster typical of other nitrogen fixers, as well as most nitrogen-dependent regulation genes. Iron oxidation in P3G is predicted to proceed through an outer membrane, high-molecular-mass cytochrome *c*, a periplasmic cupredoxin, and other mono- and dihemic cytochromes, resembling the well-studied *Acidithiobacillus ferrooxidans* iron oxidation pathway (8). The cognate COX terminal oxidase involved in iron oxidation is absent but might be replaced by a high-oxygen-affinity terminal oxidase of the *cbb*_3_ type, clustering together with a predicted high potential iron-sulfur protein and a molybdopterin oxidoreductase, inferred to play a role in electron transport during Fe(II) oxidation in *Mariprofundus ferrooxydans* PV-1 (9), and possibly also in other iron-oxidizing beta- and zetaproteobacteria. Genes encoding a *bc*_1_ complex, potentially involved in uphill electron transfer, are also present. Additionally, P3G has several hydrogenase-encoding genes that may confer hydrogen oxidation capacity. Despite being described as nonmotile, a complete suite of genes for flagella formation and chemotaxis were identified. “*F. myxofaciens*” P3G central carbon metabolism pathways include a complete glycolysis and tricarboxylic acid (TCA) cycle, suggesting that it may be a facultative heterotroph.

### Nucleotide sequence accession numbers.

This whole-genome shotgun project has been deposited at DDBJ/EMBL/GenBank under the accession no. JPOQ00000000. The version described in this paper is the first version, JPOQ01000000.
